# The effects of food stoichiometry and temperature on copepods are mediated by ontogeny

**DOI:** 10.1007/s00442-018-4183-6

**Published:** 2018-06-13

**Authors:** Lauren Mathews, Carolyn L. Faithfull, Petra H. Lenz, Craig E. Nelson

**Affiliations:** 1 Center for Microbial Oceanography: Research and Education, Department of Oceanography and Sea Grant College Program, University of Hawai‘i at Mānoa, Honolulu, USA; 2 Department of Ecology and Environmental Sciences, Umeå University, Umeå, Sweden; 3 Gävleborg County Administrative Board, Gävle, Sweden; 4 Pacific Biosciences Research Center, University of Hawai‘i at Mānoa, Honolulu, USA

**Keywords:** Nauplii, Warming, Food web, Metabolism, Zooplankton, Phosphorus

## Abstract

Climate change is warming the oceans, increasing carbon dioxide partial pressure and reducing nutrient recycling from deep layers. This will affect carbon (C) and phosphorus (P) availability in the oceans, thus, altering the balance between the nutrient content of consumers and their food resource. The combined effects of food quality and temperature have been investigated for adult copepods; however, nauplii, the early developmental stages of copepods, often far outnumber adults, grow more rapidly and have a higher phosphorus body content and demand than later life stages. Consequently, ontogeny may affect how copepods respond to the combined stressors of increasing temperature and altered food stoichiometry. We conducted temperature-controlled experiments (24, 28 and 32 °C) where *Parvocalanus crassirostris* was fed either a P-replete or a P-limited phytoplankton food source. Reduced survival of nauplii and copepodites at the highest temperature was ameliorated when fed P-replete food. At higher temperatures, copepodite growth remained stable, but internal C:P stoichiometry diverged in the direction of phytoplankton C:P, suggesting that increased temperature affected copepodite stoichiometric homeostasis. In contrast, naupliar P content increased with temperature and naupliar growth was P limited, suggesting nauplii required additional phosphorus at higher temperatures. We conclude that resource stoichiometry plays a key role in how copepod survival and growth are impacted by temperature, and that ontogeny mediates these responses. Our results suggest that as the extent of warming oceans and phytoplankton nutrient limitation increase, copepod survival and the growth of early life stages may decline.

**Electronic supplementary material:**

The online version of this article (10.1007/s00442-018-4183-6) contains supplementary material, which is available to authorized users.

## Introduction

Climate change will challenge organisms to adapt to new environmental conditions. Warming of the earth’s atmosphere and increased carbon dioxide availability are two trends we see today that may change the chemical composition and growth rates of autotrophs (Urabe et al. 2003; Riebesell et al. 2007; Toseland et al. 2013). In the ocean, warmer surface temperatures will lead to stronger stratification and decreased P recycling from the deep ocean, and increased partial pressure of carbon dioxide increases the availability of carbon relative to other nutrients (Riebesell et al. 2007; Thomas et al. 2013; Karl 2014). Phytoplankton chemical composition tends to be more flexible than their consumers’ body stoichiometry and more closely reflects the availability of carbon and nutrients (Urabe et al. 2003; Klausmeier et al. 2004; Meunier et al. 2016). In contrast, consumers are generally more nutrient rich than phytoplankton, have rigid metabolic needs, and regulate key physiological processes such as uptake, incorporation, and release of elements, to adjust for the mismatch between their body stoichiometry and the elemental content of their food source (Elser et al. 2003; Brown et al. 2004; Frost et al. 2005; Riebesell et al. 2007). Therefore, climate change may challenge consumers to adapt to a future food resource that may not chemically reflect the food source they rely on today, or be outcompeted by species with optimized nutrient uptake affinities, storage and retention traits (Meunier et al. 2016).

Numerous animals undergo dramatic shifts in ecology over their life history, presumably to maximize growth, survival and reproduction of the organism over their life span. Warmer oceanic temperatures may affect timing and duration of consumer developmental stages, metabolic rates and nutrient demand. Copepods are an important phytoplankton consumer group (Taylor et al. 2012), which undergo a metamorphosis between the 6th nauplius and 1st copepodite stage, which includes rearrangement of appendages and nervous system, shifts in feeding behavior, nutrient demand and body stoichiometry (Paffenhöfer and Lewis 1989; Peterson 2001; Titelman and Kiørboe 2003; Wilson and Hartline 2011). Due to the abundance of copepods and especially nauplii in the ocean (Turner 2004), how copepod stages respond differentially to warming and changes in phytoplankton stoichiometry will have implications for nutrient cycling and food web dynamics (Frost et al. 2005; Riebesell et al. 2007).

Two main hypotheses describe how herbivore stoichiometry and nutrient demand should respond to temperature: (1) the growth rate hypothesis, and (2) the metabolic efficiency theory. The growth rate hypothesis argues that nutrient demand should increase with temperature due to higher growth rates and associated increased phosphorus demands for RNA and protein synthesis (Elser et al. 2003; Vrede et al. 2004). The metabolic efficiency theory argues that as temperature increases the efficiency of RNA and protein synthesis in biochemical reactions increases; therefore, higher growth rates can be maintained with fewer nutrients at higher temperatures (McFeeters and Frost 2011; Toseland et al. 2013). Recent studies have found that adult copepods seem to be less affected by nutrient limitation with increasing temperature, suggesting their nutrient use becomes more efficient at higher temperatures in accordance with the metabolic efficiency theory (Malzahn et al. 2016; Anderson et al. 2017). However, due to the dramatic physical and stoichiometric differences between nauplii and copepodite stages, ontogeny may influence zooplankton responses to temperature and food stoichiometry. For example, a recent study found that nauplii growth was more sensitive to food nutrient content than adult copepods (Bullejos et al. 2014). Our study addresses the interactive effects of food quality and temperature on the survival, growth, stoichiometry and grazing rate of both naupliar and copepodite stages of the widespread subtropical calanoid copepod species, *Parvocalanus crassirostris*.

Due to higher growth rates and the higher P demand of nauplii relative to copepodites (Elser et al. 2003; Bullejos et al. 2014; Meunier et al. 2016), we hypothesize that nauplii will follow the predictions of the growth rate hypothesis, with naupliar phosphorus demand increasing with warmer temperatures (Elser et al. 2003; Persson et al. 2011). As copepodites are slower growing and nearing reproduction, their survival may be maximized by adapting to higher temperatures and P limitation using phosphorus more efficiently according to the metabolic efficiency theory (Cross et al. 2015; Boersma et al. 2016). Because of the abundance and importance of *P. crassirostris* as a larval fish food source (Sampey et al. 2007; Kline and Laidley 2015), changes in the survival, growth, and stoichiometry of this copepod species with climate change may alter subtropical coastal food webs. This is the first time the contrasting predictions of the growth rate hypotheses and the metabolic efficiency theory have been tested in the same organism at different life history stages. Our results will lead to a better understanding of how ontogeny influences consumer phosphorus demand in response to warming.

## Materials and methods

**Fig. 1:**
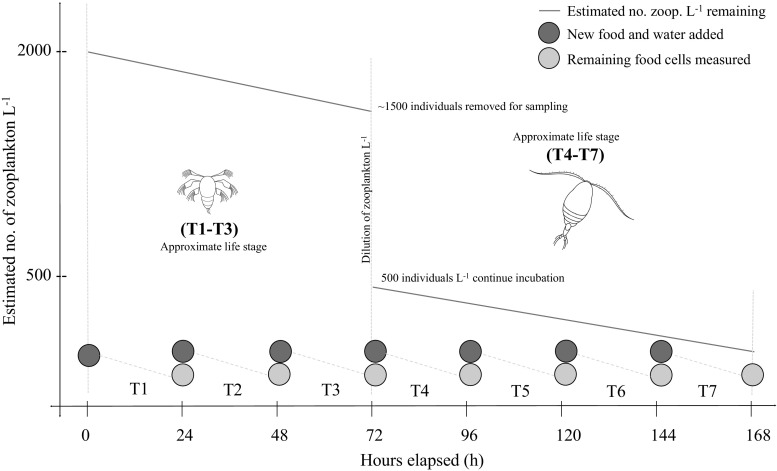
Sampling regime schematic illustrating (1) when the nauplii (hour 0 and 72) and copepodites (hour 168) were collected for sampling and which measurements were taken, (2) the estimated number of zooplankton L^−1^ surviving through the 168-h (7 day) incubation including the removal of approximately 1500 zooplankton L^−1^ at hour 72 which yielded a remaining 500 zooplankton L^−1^ in incubation bottles at the start of T4, and (3) the regimen of the complete replacement of food every 24 h

To investigate how temperature and food phosphorus (P) content affect life history parameters (survival, growth rate, grazing rate and carbon:phosphorus stoichiometry) of the calanoid copepod *P. crassirostris*, we conducted three experiments at 25, 28, and 32 °C to simulate average winter (24–25 °C), summer (26–28 °C) and extreme summer sea surface temperatures in a tropical coastal environment (Jokiel and Brown 2004; Thomas et al. 2013). Experiments were based in Hawai’i and took place during 19–25 Nov 2015, 11–16 Mar 2016 and 14–20 Jan 2016 for the 25 °C, 28 °C and 32 °C experiments, respectively. In each experiment, *P. crassirostris* were fed *Tisochrysis lutea* cultures grown at two different nutrient stoichiometries (P replete and P limited) for 7 days, spanning one life cycle from nauplius to adult. For *P. crassirostris*, it takes approximately 3–4 days to reach the CI stage, and then another 3–4 days from CI to CVI. To capture the variation between naupliar and copepodite stages, each 7-day experiment was divided into two parts, where the first part (day 0–day 3) captured the life history information for nauplii (~ 2000 ind L^−1^), which were then sampled on day 3, and rediluted to 500 L^−1^ for the second part of the experiment (day 4–day 7), which provided life history information for copepodites (Fig. 1). The timing of the two sampling occasions, the first after 3 days to capture the naupliar stages and the second after a further 4 days, ensured that a proportion of copepodites would reach adulthood, but there would not be much egg production.

### Phytoplankton culture

For both cultures and experiments sub-surface seawater was collected from Kāneʻohe bay, O‘ahu, Hawaiʻi (21°27′31.7″N 157°48′03.5″W) and filtered sequentially through glass fiber (0.7 μm pore size GF/F) and 0.1 μm polycarbonate filters. The filtered seawater (FSW) was kept cool (4 °C) until 24 h before the start of each experiment when it was placed in an incubator at the experimental temperature (25, 28, or 32 °C). To create the two food treatments, *T. lutea* was cultured under two different nutrient and light conditions. For the P-replete treatment, *T. lutea* was cultured using P-replete f/2 medium (Andersen 2005) and a light level of 17.6 ± 1.32 μmol m^−2^ s^−1^. For the P-limited culture, *T. lutea* was cultured in f/2 media without phosphate and at a higher light level (60.1 ± 3.36 μmol m^−2^ s^−1^). The *T. lutea* cultures were incubated in batch culture in 6 × 500-mL sterile polycarbonate cell culture flasks, with one flask allocated to each day of the experiment. To control for potential variation in phytoplankton cultures over time, each flask was inoculated from an axenic *T. lutea* stock culture 2 weeks prior to its use during the experiment to allow the cultures to achieve a cell density above 9 × 10^5^ cells mL^−1^, and to ensure that every day the copepods received fresh algae from a new bottle in the same growth phase (Fig. 1). C, N and P samples from the phytoplankton cultures fed to the zooplankton were measured daily to control for any variation. All cultures were incubated at a 12:12, light:dark cycle at 25 °C. P-replete cultures had an average C:P of 141:1 ± 46 (mean ± standard deviation), whereas P-limited cultures had an average C:P of 1158 ± 134. Stoichiometry of the *T. lutea* fed to the zooplankton cultures over time is presented in Online Resource Fig. S2.

### Copepod culture

*Parvocalanus crassirostris* stock cultures have been continuously maintained in 20-L polycarbonate containers at room temperature (approximately 25–27 °C) at the Pacific Biosciences Research Center in the Lenz lab since 2008. To obtain a fresh cohort of nauplii for each experiment, we removed approximately 10,000 adults by filtering the source culture through a 162-μm mesh filter and gently washing adults caught on the mesh into a separate 10 L (FSW) egg-laying culture 20 h before the start of each experiment. The adults were fed 40 mL of *T. lutea* (mean density of 9 × 10^5^ cells mL^−1^) per 1 L of seawater to promote egg production. After 15 h, adults were removed by filtering the egg-laying culture three times using a 162-μm filter. The remaining culture contained only nauplii and eggs produced during the overnight egg-production incubation. Nauplii were then filtered onto a 20-μm filter and washed into fresh FSW. This was repeated three times to obtain approximately 36,000 nauplii between the NI–NIII stages for each experiment.

### Experimental methods

On day 0, 2000 NI–NIII nauplii (6–18 h old) were added to six of the twelve 1 L treatment bottles. Naupliar densities in the egg production containers were estimated by counting nauplii in three subsamples, and then adding the appropriate volume to achieve a population of 2000 animals per experimental bottle. Three bottles with nauplii and three bottles without nauplii received the P-limited *T. lutea* culture and the same for the P-replete *T. lutea.* After every 24 h period, the animals were transferred into fresh food suspensions. The daily dosage of food for both treatments was equivalent to 0.1 µg C L^−1^ day^−1^, which was found to be not limiting for *P. crassirostris* growth at the experimental densities in an earlier pilot study and by Böttjer et al. (2010). Culture density of *T. lutea* was determined by making triplicate cell counts using a double Neubauer counting chamber and viewed under a Leica compound microscope (200× magnification). *T. lutea* culture cell counts were converted to carbon units using the method of Menden-Deuer and Lessard (2000):

$$\log_{10} {\text{C}} \left( {\text{pg}} \right) = -\, 0.541 + 8.11 \times \log_{10} \left( V \right).$$

Additionally, two 20 mL subsamples from each *T. lutea* culture were filtered onto acid-washed (1.5 M HCl) and combusted (510 °C, 4 h) glass fiber filters (Whatman, GF/F) and immediately frozen at − 20 °C. One filter was used to measure particulate carbon (C) and nitrogen (N) and the other particulate phosphorus (P).

Experimental bottles were incubated in the dark on a plankton roller for 7 days at the experimental temperature (experiment 1: 25 °C, experiment 2: 28 °C, and experiment 3: 32 °C). After each 24 h period of incubation, each bottle was gently filtered through a 20 μm filter, and the animals carefully transferred into an acid-washed 1 L polycarbonate bottle with FSW. After the appropriate food was added to each treatment bottle, bottles were returned to the dark incubator. From the filtrate remaining after the animals were removed, 4 mL was preserved for flow cytometric analysis with 200 μL of 8% paraformaldehyde and frozen at − 80 °C within 30 min of sampling, to determine remaining cell densities and calculate grazing rates.

Additional sampling to measure naupliar and copepodite survival, biomass and stoichiometry was conducted on day 0, day 3 and day 7, respectively (Fig. 1). To measure naupliar survival from day 0 to day 3 and copepodite survival from day 3 to day 7, animals from each bottle were concentrated to 100 mL and the numbers of individuals in five subsamples of 2 mL volumes were counted. On day 3, 500 individuals of the original 2000 animals from each replicate per treatment were transferred into a new treatment bottle and were incubated for the remaining four 24 h periods. The remaining animals (ca. 1500) were divided for biomass and stoichiometric measurements. On day 0, day 3 and day 7, 20–50 animals were first killed with 95% ethanol and then preserved in 70% ethanol for biomass measurements. Stoichiometry was measured on the remaining animals (from the treatment bottles on day 3 and day 7, and from the egg production culture on day 0), which were placed in FSW for approximately 2 h before being washed onto an 80 μm mesh filter three times to remove food cells, and then onto a 20 μm mesh filter, where they were first rinsed three times and then stored in Milli-Q water. Samples were frozen at − 80 °C for later analysis of particulate carbon, nitrogen and phosphorus.

To estimate biomass, we measured the stage and total length or prosome length, for nauplii and copepodites, respectively, at 60× magnification using a Leica light microscope. Lengths of each recorded developmental stage within a sample were averaged and an average dry weight in micrograms for each stage was calculated using the life-stage-specific length–dry weight regressions for *P. crassirostris* as in Hopcroft et al. (1998). These data were used to determine copepod stage distributions. Due to inconsistencies in dry weights obtained using the vacuum concentration method (see below for method), we were unable to derive a confident value for zooplankton dry weight. Consequently, to estimate carbon content we used dry weights obtained from length–dry weight regressions and converted to C content using a C dry weight^−1^ ratio of 0.45 (Omori and Ikeda 1984). The percentage of each stage counted and the survival data were used to calculate the average dry weight per individual for each treatment on day 0, day 3 and day 7. Specific growth rates (*G*) were calculated from the average dry weights of individuals on day 0, day 3 and day 7, using: *G* = ln(*W*_*t*_/*W*_0_)/_*t*_, where *W*_t_ is the weight at the end and *W*_0_ is the weight at the beginning of time period *t*.

### Elemental analysis

Before analysis of carbon and nitrogen, the zooplankton samples were vacuum concentrated (SAVANT ISS110, Thermo Scientific) and weighed in tin capsules. Samples of *T. lutea* preserved on glass fiber filters were dried at 60 °C, and packaged into tin capsules. All carbon and nitrogen measurements were made via high-temperature combustion on an Exeter Model CE 440 elemental analyzer at the SOEST laboratory for analytic biogeochemistry (University of Hawai’i at Mānoa). Particulate carbon and nitrogen standards were prepared using acetanilide (C_8_H_9_NO; molecular weight = 135.16). For analysis of particulate phosphorus, the freeze-dried zooplankton samples were weighed, and zooplankton and filters were placed in individual acid-washed and pre-combusted glass combustion tubes, before combusting at 510 °C for 4 h to convert organic phosphorus to orthophosphate. For each sample, 10 mL of 0.15 M HCl was added, then vortexed and centrifuged before determination of the liberated orthophosphate by colorimetry following the procedure outlined in Strickland and Parsons (1972). Due to inconsistencies in dry weights obtained using the vacuum concentration method we were unable to calculate specific C, N and P contents of zooplankton. Instead, here, we present molar ratios (e.g., C:P), which are independent of dry weight.

### Grazing rate

To compare grazing rates among treatments, we quantified the number of live *T. lutea* cells remaining after each 24 h incubation using flow cytometry. Following thawing from − 80 °C, 20 µL of each sample was subsampled into a 96-well plate and stained with SYBR green (1× final concentration). The plate was run on an Attune Flow cytometer, and *T. lutea* were categorized to be distinct populations of events characterized by high side scatter and forward scatter, high chlorophyll *a* and high DNA (SYBR green) content. Events that satisfied all of four conditions were quantified into a concentration of event µL^−1^. Control treatment bottles were analyzed to account for changes in cell densities due to natural cell death or growth, and changes in cell counts in bottles without zooplankton for each food treatment were subtracted from each paired zooplankton bottle. Negative grazing rates were corrected to zero. Grazing rate was calculated as *T. lutea* cells consumed per mg C of naupliar (day 0–day 3) or copepodite biomass (day 4–day 7) per day (cells mg C^−1^ day^−1^) (Moorthi et al. 2016). To estimate the number of zooplankton in each bottle at each time point we assumed that animal mortality occurred at a constant rate between the time points where animal density data were available (day 0, day 3, day 7).

### Statistical analyses

Data were examined for normality and heteroscedasticity of variances using the Shapiro–Wilks test of normality, and then log_10_ transformed to meet model assumptions of normal distribution and heteroscedasticity of variances. We used two-way analysis of variance (ANOVA) with an interaction term to determine the responses of growth, average dry weight, and body stoichiometry to temperature, food treatment, and their interaction. Tukey’s honestly significant differences were performed on all ANOVA results; to avoid clutter, only significant *P* values and interactions are indicated in the graphs. To determine the effect of temperature and food treatment on daily grazing rates, we used repeated measures ANOVA, which was adjusted using covariance partitioning and model selection to account for the interdependence within bottles over time according to the methods outlined in Zuur et al. (2009), using the nlme package (Pinheiro et al. 2017). Survival data were analyzed as percentage survival using a linear model fitted with generalized least squares. An autoregressive correlation structure was used to describe the within-group correlation structure. This correlation structure was determined following the methods outlined in Zuur et al. (2009), by comparing different correlation structures using the Akaike and Bayesian information criterions and log likelihood to find the simplest model which best fit the data. To determine if stage distributions differed with temperature and food treatment, we used a paired Kolmogorov–Smirnov non-parametric test. All statistical analyses were performed using the R Statistical Programme (3.3.1).

## Results

**Fig. 2:**
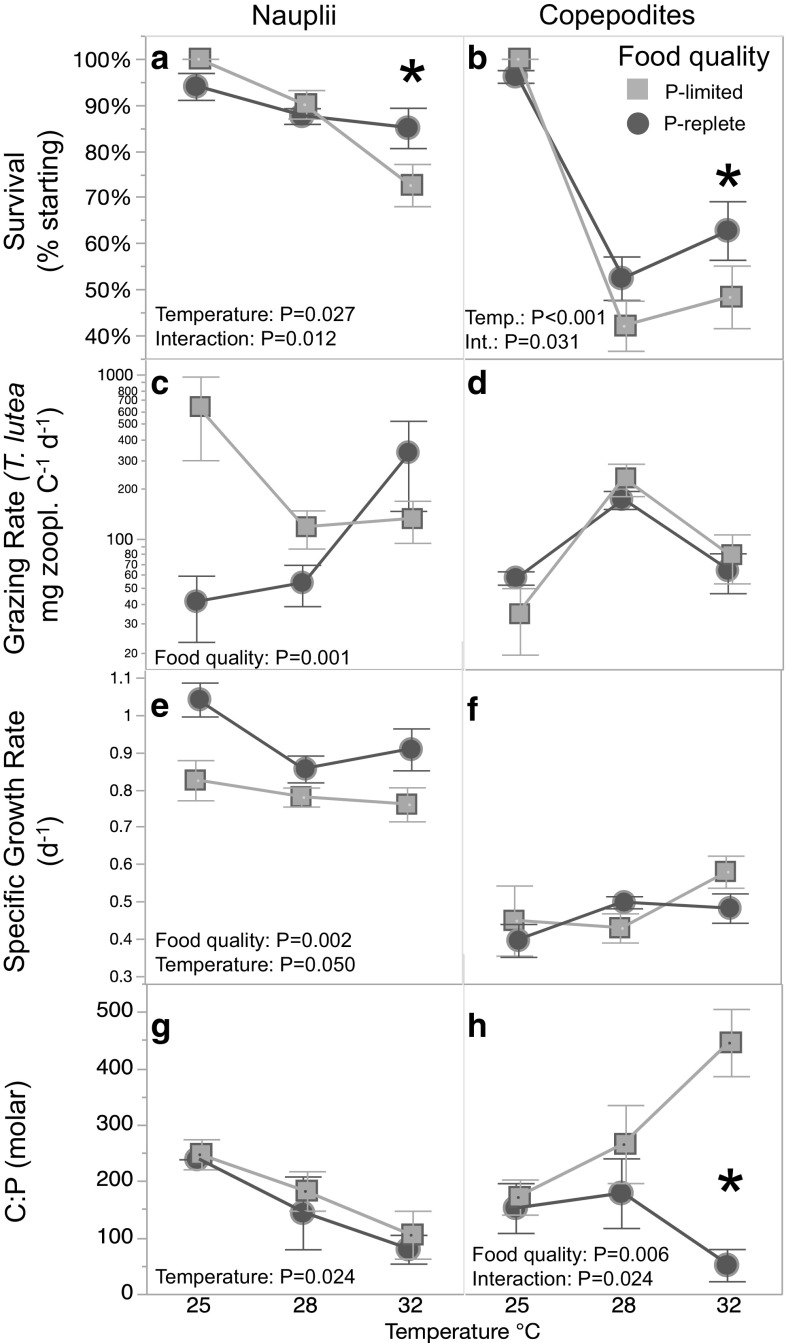
Life history traits of the copepod *P. crassirostris* after 3 days (nauplii, left column) and 7 days (copepodites, right column) when fed P-limited (light) and P-replete (dark) phytoplankton at each of the three incubation temperatures: 25, 28, and 32 °C. All values are mean ± 1 standard error of the mean of three replicate incubation bottles (*N* = 3). Each panel is annotated with significant *P* values from two-way ANOVA. Significant post hoc interactions between food quality at specific temperatures are represented by *. **a**, **b** The percentage of copepods surviving; **c**, **d** the specific copepod grazing rate (cells of *Tisochrysis lutea* zooplankton mg C^−1^ h^−1^); **e**, **f** the specific growth rate (day^−1^); **g**, **h** are the internal body stoichiometry of carbon:phosphorus (molar) for nauplii and copepodites, respectively

Temperature had the greatest effect on survival, with both nauplius and copepodite survival decreasing with temperature. Average survival was higher for nauplii (mean ± standard error: 88 ± 4%) over the first 3 days, than for copepodites, 67 ± 10%, over the latter 4 days of each experiment. For nauplii, survival was 19% lower at 32 °C than at 25 °C (Fig. 2a). Copepodite survival was also highest at 25 °C, being twice as high compared with survival at 28 or 32 °C (Fig. 2b). The C:P ratio of P-limited food (1158 ± 134) was eight times higher than P-replete food (141 ± 46). Stoichiometry of the *T. lutea* fed to the zooplankton cultures over time is presented in Supplementary Information Fig. S2. The P content of the food did not affect nauplii or copepodite survival at 25 or 28 °C. However, at 32 °C when fed P-replete food, nauplii survival was 15% higher and copepodite survival increased by 23% compared with those fed P-limited food.

Both nauplii and copepodites were heavier when fed P-replete food. Naupliar-specific dry weight was 37% higher when fed P-replete food than P-limited food, and nauplii were 30% heavier at 25 °C than at 28 °C or 32 °C (*F*_2,12_ = 16.9, *P* = 0.001). Copepodites fed P-rich food had 25% higher specific dry weight across all three temperatures (*F*_2,12_ = 12.6, *P* = 0.004). There was a borderline effect of temperature on copepod biomass with copepodites at 32 °C being 24% heavier than copepodites at 28 °C (*F*_2,12_ = 3.83, *P* = 0.052). There was no significant interaction between the effects of temperature and food treatment on nauplius or copepodite average specific dry weight.

The average specific grazing rate for nauplii was 248 ± 81 (mean ± SE) cells of *T. lutea* zooplankton mg C^−1^ h^−1^. Grazing rates for copepodites averaged 93 ± 12 cells of *T. lutea* zooplankton mg C^−1^ h^−1^. Grazing rates were below detection rates (i.e., zero) at 40% of sampling occasions for nauplii and 24% of sample occasions for copepods. Phytoplankton stoichiometry had a significant effect on naupliar grazing rate, where nauplii fed P-limited phytoplankton had 18 times as high grazing rates than nauplii fed P-replete food (Fig. 2c). Copepodite grazing rate did not change with food stoichiometry or temperature (Fig. 2d).

The average specific growth rate for nauplii was 0.86 ± 0.11 d^−1^ and was affected by food quality and temperature. Naupliar-specific growth rate was nearly 20% higher when nauplii were fed P-rich food. In addition, naupliar-specific growth rates were highest at 25 °C (Fig. 2e). There was no interaction between temperature and food treatment on naupliar growth rate. The average specific growth rate for copepodites was 0.47 ± 0.10 d^−1^. Copepodite growth rates did not differ with food P content and did not differ significantly with temperature (Fig. 2f).

Naupliar internal C:P and C:N were on average lower than copepodite internal stoichiometry, except at 25 °C where naupliar and copepodite stoichiometry did not differ significantly. Naupliar internal C:P averaged 189 ± 8.72 (mean ± SE) and C:N 10.1 ± 1.07. Temperature had a negative effect on naupliar C:P (Fig. 2g). This was due to the P content of nauplii increasing with warmer temperatures (*F*_2,12_ = 4.47, *P* = 0.035), while naupliar internal C concentrations did not change with temperature (*F*_2,12_ = 0.729, *P* = 0.503). Elemental content of nauplii did not change with food quality; however, food quality had a large effect on copepodite stoichiometry. Average copepodite C:P was 242 ± 11.3 (mean ± SE) and copepodite C:N was 17.0 ± 3.11. Differences in copepod stoichiometry were due to changes in P with food treatment and changes in C content were not significant. Copepod internal C:P did not differ with temperature alone, instead the effect of the different food treatments on copepodite internal C:P interacted with temperature. At 25 °C, copepodites maintained a steady C:P ratio regardless of food P content. In contrast, at 32 °C, copepodite body C:P diverged with P content of the food, and was nearly 11 times higher when fed the P-limited than when fed P-replete phytoplankton (Fig. 2h).

## Discussion

The role of ontogeny in influencing fundamental life history traits is a central tenant of biology, and these experiments sought to investigate the influence of ontogeny on how the phosphorus requirement of naupliar and copepodite stages changes with increasing temperature. During development, copepods undergo a major transformation between the naupliar and copepodite stages. Between the last nauplius (NVI) and the first copepodite (CI) stage there is high mortality (Peterson 2001) associated with major morphological modifications, which include changes in swimming and feeding behavior, changes in the sensory system, and a reorganization of the nervous system (Paffenhöfer and Lewis 1989; Peterson 2001; Wilson and Hartline 2011). These changes have stoichiometric implications: body carbon-specific ingestion rates of nauplii are high and typically exceed 100% of body C per day (Meyer et al. 2002; Henriksen et al. 2007; Jungbluth et al. 2017), while those of adult copepods are below 30% of body C per day (Meyer et al. 2002). Furthermore, C:N ratios are lower in nauplii than copepodites (Campbell et al. 2001). Thus, the growth requirements for nauplii differ from those of copepodites, which in turn could lead to higher physiological sensitivity to low nutrients as has been reported in previous work (Bullejos et al. 2014).

We hypothesized that nauplii would follow the predictions of the growth rate hypothesis, with naupliar phosphorus demand increasing with warmer temperatures, as found for freshwater zooplankton by Persson et al. (2011) and Wojewodzic et al. (2011). With increasing temperature, nauplii became more P rich, naupliar grazing rate on P-rich food increased, and naupliar survival was higher when fed P-replete food, all findings are consistent with the growth rate hypothesis (Elser et al. 2003). In contrast, we hypothesized that as copepodites have lower specific growth rates and are nearing reproduction, their survival may be maximized by using phosphorus more efficiently according to the metabolic efficiency theory (McFeeters and Frost 2011; Cross et al. 2015). We found patterns that support the metabolic efficiency theory for copepodites, whereby when copepodites were fed high-C:P food, growth was not P limited at the highest temperature and copepodite C:P increased with temperature. However, it should be noted that phosphorus rich food increased copepod survival at the highest temperature—which disagrees with the metabolic efficiency theory. Thus, it appears that these two competing models of nutrient limitation may apply to some degree in the same organism at different stages of their lifecycle. Below we discuss how ontogeny may mediate the effects of food stoichiometry and temperature on *P. crassirostris* survival, grazing, growth and C:P stoichiometry.

Survival of nauplii and copepodites was negatively affected by higher temperatures. 25 and 28 °C are well within the natural range experienced by *P. crassirostris* in coastal Hawaiian ecosystems (Bahr et al. 2015). However, 32 °C is 3 °C higher than the average summer surface temperature, but close to the maximum observed coastal summer temperatures in Hawai‘i (Thomas et al. 2013), and approximates local thermal maxima during recent bleaching events in Kāne‘ohe Bay (Bahr et al. 2015). We found that the number of nauplii and copepodites each bottle could support declined with increasing temperature. In accordance with the growth rate hypothesis, Brown et al. (2004) argued that as temperatures rise the number of individuals a specific environment can support will decline, as the same number of individuals will recycle carbon and nutrients at a higher rate. For this relationship to hold, there must be a limited amount of resources available and carbon or nutrients must limit growth at higher temperatures. The food concentrations used in this experiment were not found to be limiting for *P. crassirostris* in an earlier pilot study or by Böttjer et al. (2010). Although neither of these studies tested food limitation at 32 °C, there were still phytoplankton cells left in the bottles after 24 h, and it is unlikely that C limited growth at 32 °C, as survival was higher when fed P-replete food at 32 °C. Indeed, we found that when resources were higher in terms of nutrient availability, as in the P-replete treatments, both nauplii and copepodites had higher survival rates at 32 °C. This suggests that higher phosphorus availability partly reduced the extra stress placed on the organisms by warmer temperatures. Lower survival at higher temperatures could also be due to faster developmental times and, therefore, earlier mortality (Rhyne et al. 2009; Malzahn et al. 2016). In our experiment, individuals did not age faster in warmer temperatures or in the P-replete treatments; therefore, the effects of temperature and food quality on survival and growth rates were not due to changes in developmental times or population distributions (Online Resource, Fig. S1).

We predicted that copepodite and naupliar stages would respond differently to temperature and phosphorus availability. Naupliar growth rate was higher with P-replete food across all temperatures and copepodite growth was not dependent on food P content. Sensitivity to nutrient mineral constraints may decrease with ontogeny in copepods, with nauplii having lower C:nutrient ratios than adult copepods and being more prone to nutrient limitation than older stages (Laspoumaderes et al. 2010; Bullejos et al. 2014). In our study, naupliar C:P ratios were not consistently lower than those of copepodites; however, we found that naupliar C:P internal stoichiometry did not change with the C:P content of their food, whereas copepodite internal stoichiometry reflected their food source at higher temperatures. Instead, nauplii became more phosphorus rich at higher temperatures, suggesting that naupliar phosphorus use did not become more efficient with warming. This is thought to be due to nauplii having higher specific growth rates and, therefore, higher phosphorus demands for ribosome and RNA production (Elser et al. 2003; Vrede et al. 2004; Meunier et al. 2016). With rising ocean temperatures, nauplii are likely to become even more phosphorus limited since their body phosphorus requirement will increase.

For copepodites, there was a decoupling of growth and survival in our study: phosphorus availability enhanced survival at higher temperatures but did not increase copepodite growth rates. If copepods need greater amounts of phosphorus at higher temperatures to cope with increased RNA and phosphorus demands (i.e., Elser et al. 2003; Persson et al. 2011), we would expect growth rates to be highest in the P-replete treatment when temperature is highest. Instead, we found copepodite growth rate at 32 °C was similar in both low and high C:P treatments. At higher temperatures, copepods may require more carbon to cope with higher metabolic demands due to increased respiration relative to growth (Boersma et al. 2016; Malzahn et al. 2016). Thus, the patterns of copepodite growth in our experiment follow the metabolic efficiency theory; copepodite growth was uncorrelated with the C:P ratio of phytoplankton across all temperatures (Fig. S3), and growth was higher at 32 °C when more carbon was available relative to phosphorus (Cross et al. 2015; Boersma et al. 2016). Consistent with the metabolic efficiency theory, copepodite body C:P increasingly diverged to reflect the food source (either P-replete or P-limited) as temperature increased.

Past studies suggest that the nitrogen and phosphorus content of organisms generally declines with increasing temperature (Rhee and Gotham 1981; Woods et al. 2003). The larger size and slower relative growth of copepodites may have enabled them to reallocate elements and have a more flexible body stoichiometry than nauplii. As temperature increases, the efficiency of RNA and protein use in biochemical reactions increases and the quantity of proteins needed for growth decreases according to the metabolic efficiency theory (McFeeters and Frost 2011; Toseland et al. 2013). Copepodite growth rates did not differ with food quality at 32 °C, even as internal C:P stoichiometry increased when fed P-limited food; therefore, it appears that copepodites used phosphorus more efficiently at higher temperatures (Woods et al. 2003; McFeeters and Frost 2011; Cross et al. 2015).

To obtain nutrients required for growth and development, zooplankton can select higher quality food (selective grazing), or graze at higher rates on low-quality food (compensatory grazing) (Hillebrand et al. 2009; Meunier et al. 2016). In our experiment, naupliar grazing rates were highest when fed P-limited food across all temperatures, which may be a compensatory mechanism to obtain the phosphorus required for growth, while respiring or excreting the excess carbon (Hillebrand et al. 2009). In our experiment, naupliar growth was P limited across all temperatures, which suggests that nauplii were unable to completely compensate for lower food quality by increasing their grazing rate on P-limited food (Moorthi et al. 2016). Copepodites did not exhibit compensatory grazing, which may reflect their lower nutrient demand (Bullejos et al. 2014; Meunier et al. 2016). Copepodite grazing rate did not increase with temperature (Brown et al. 2004; Hillebrand et al. 2009; Moorthi et al. 2016). Instead copepodites had the highest grazing rates at 28 °C, which represents summer sea surface temperatures in coastal Hawai’i waters (Bahr et al. 2015). The lower survival and reduced homeostasis of copepodite internal stoichiometry at 32 °C suggest that this temperature is outside the optimal environmental range for *P. crassirostris* in Hawai’i.

There was no significant effect of temperature on zooplankton biomass in either ontogenetic stage. The lack of temperature effect on naupliar- and copepodite-specific body mass emphasizes that temperature has a greater effect on more dynamic life history parameters such as survival and grazing rates, while zooplankton maintain a stable mass dictated largely by their available nutrient resources. This was evident in the positive effect that P-replete food had on both naupliar and copepodite individual biomass. Decreased nutrient availability may result in reduced metazoan body sizes due to suboptimal protein synthesis, which is linked to growth rate and development (Sterner 1993; Elser et al. 2003; Cross et al. 2015). Characteristics of nauplii, such as high growth rates, high phosphorus demand and small body size, may have contributed to increasing phosphorus requirements at warmer temperatures according to the predictions of the growth rate hypothesis. In contrast, copepodite phosphorus demand appeared to decline with warming, in accordance with the metabolic efficiency theory; however, copepodite survival was still positively affected by phosphorus availability at extreme temperatures. These results, although only for a single species, illustrate that ontogeny has important implications for carbon and phosphorus demand with temperature. Young fast-growing stages may depend more on limiting nutrients (Bullejos et al. 2014) than slower growing older stages, which have greater nutrient reserves to draw upon when exposed to stressful nutrient-limiting conditions. Further investigation into the cellular mechanisms behind why adult copepods and not nauplii are able to employ more efficient use of phosphorus at higher temperatures may reveal important metabolic insights as to why organisms exhibit ontogenetic shifts.

Due to the abundance and relatively high grazing impacts of nauplii (Jungbluth et al. 2017), nauplii may have a different and significant influence on carbon and nutrient cycling compared to adult and copepodite stages. Due to the decreasing C:P stoichiometry and increasing phosphorus demand of nauplii with rising temperature, it is possible that abundant nauplii may reduce phosphorus recycling in an already P-limited ocean. Copepodites, on the other hand, are unlikely to alter P recycling in a warming ocean due to their flexible C:P body stoichiometry and lower phosphorus demand. Future investigation is still needed to understand how copepods will affect nutrient cycling of other important elements, such as nitrogen (e.g., Meunier et al. 2016), over their lifecycles, and the feedbacks this will have on copepod population dynamics and ocean elemental cycling remain to be studied.

## Electronic supplementary material

Below is the link to the electronic supplementary material.

Supplementary material 1 (PDF 182 kb)
