# Supplementary material for: The effects of food stoichiometry and temperature on copepods are mediated by ontogeny
Source: Oecologia. 2018 Jun 13;188(1):75–84. doi: 10.1007/s00442-018-4183-6 (PMC6096765; doi:10.1007/s00442-018-4183-6)

Electronic Supplementary Material

The effects of food stoichiometry and temperature on copepods are mediated by ontogeny

Lauren Mathews[1], Carolyn L. Faithfull[1,2,3*], Petra H. Lenz[4] and Craig E. Nelson[1]

[1]Center for Microbial Oceanography: Research and Education, Department of Oceanography and Sea Grant College Program, University of Hawai‘i at Mānoa, USA.

[2]Umeå University, Department of Ecology and Environmental Sciences, Umeå, Sweden.

[3]Gävleborg County Administrative Board, Gävle, Sweden.

[4]Pacific Biosciences Research Center, University of Hawai‘i at Mānoa, USA.

* Corresponding author: clfaithfull@gmail.com

Table S1. Two way and repeated measures analysis of variance for nauplii (day 0–day 3), showing degrees of freedom (df), F-values and P-values. Significant P-values are highlighted in bold. Carbon: phosphorus (C:P) ratio is molar. Transformation where required to meet normality assumptions are shown on the far right.

Responses	Effect	df	F	P	
Survival (%)	Temperature	1,47	21.65	**<0.001**	
	Food Treatment	1,47	1.44	0.236	
	Temperature*Food Treatment	2,47	4.95	**0.012**	
Individual Biomass (μg dry weight nauplius^{-1})	Temperature	2,12	5.32	**0.022**	
	Food Treatment	1,12	16.93	**0.001**	
	Temperature*Food Treatment	2,12	2.36	0.136	
Growth Rate (d^{-1})	Temperature	2,12	3.88	**0.050**	
	Food Treatment	1,12	15.96	**0.002**	
	Temperature*Food Treatment	2,12	1.23	0.325	
Grazing Rate (*T. lutea* cells µg C^{-1} day^{-1})	Temperature	2,47	1.95	0.169	
	Food Treatment	1,47	13.60	**0.001**	
	Time	1,47	14.88	**<0.001**	
	Temperature*Food Treatment	2,47	2.18	0.147	
C:P (molar)	Temperature	2,12	5.36	**0.024**	log_{10}
	Food Treatment	1,12	0.12	0.739	
	Temperature*Food Treatment	2,12	0.55	0.590	
P content (µg P zooplankton^{-1})	Temperature	2,12	4.47	**0.035**	
	Food Treatment	1,12	0.25	0.628	
	Temperature*Food Treatment	2,12	1.87	0.196	log_{10}

Table S2. Two way and repeated measures analysis of variance for copepodites (day 4–day 7), showing degrees of freedom (df), F-values and P-values. Significant P-values are highlighted in bold. Transformation where required to meet normality assumptions are shown on the far right.

Measured Responses	Effect	df	F	P-value	
Survival (%)	Temperature	2,65	103	**<0.001**	
	Food Treatment	1,65	1.96	0.166	
	Temperature*Food Treatment	2,65	3.67	**0.031**	
Individual Biomass (μg dry weight copepodite^{-1})	Temperature	2,12	3.83	0.052	
	Food Treatment	1,12	5.32	**0.004**	
	Temperature*Food	2,12	2.07	0.170	
Growth Rate (d^{-1})	Temperature	2,12	2.26	0.147	
	Food Treatment	1,12	0.42	0.528	
	Temperature*Food Treatment	2,12	1.40	0.285	
Grazing Rate (*T. lutea* cells µg C^{-1} d^{-1})	Temperature	2,68	0.003	0.959	
	Food Treatment	1,68	0.68	0.417	
	Temperature*Food Treatment	2,68	0.12	0.725	
	Time	1,68	6.81	**0.011**	
C:P (molar)	Temperature	2,10	0.07	0.934	$\log_{10}$
	Food Treatment	1,10	12.11	**0.006**	
	Temperature*Food Treatment	2,10	5.56	**0.024**	
P content (µg P zooplankton^{-1})	Temperature	2,11	0.55	0.594	
	Food Treatment	1,11	18.38	**0.001**	
	Temperature*Food Treatment	2,11	3.21	0.079	

Fig. S1. Population distribution graphs. The proportion of the population at each stage on day 3 (nauplii, n1-n6) and day 7 (copepodites, c1-c6), when fed P-limited (light bars) and P-replete (dark bars) phytoplankton at each of the three incubation temperatures: a) and b) 25°C, c) and d) 28°C, and e) and f) 32°C. We found no statistically significant differences in population distributions with food quality or temperature when using a paired Kolmogorov-Smirnoff non-parametric test in the R Statistical Programme.

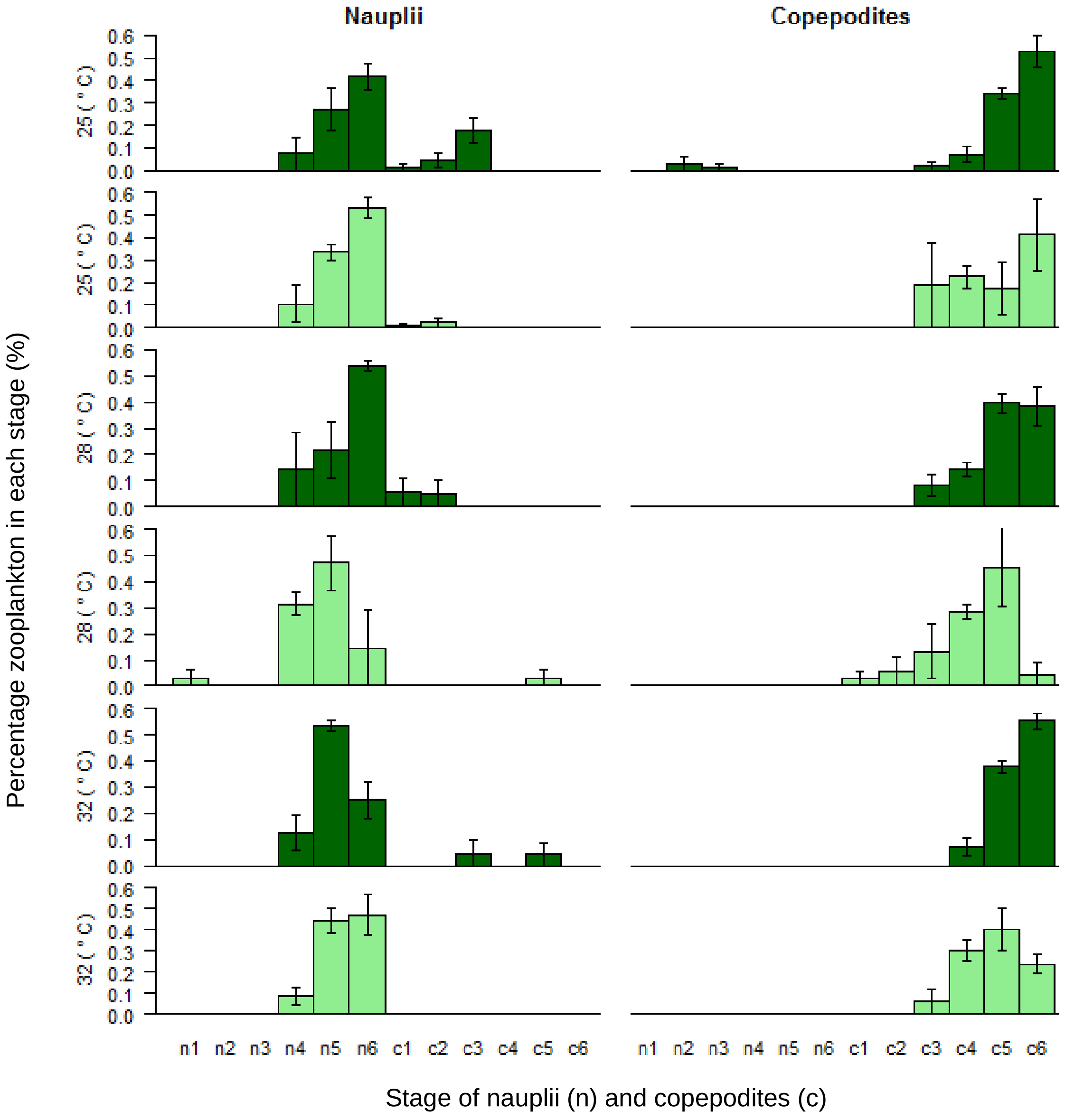

Fig. S2. The average carbon: phosphorus stoichiometry ± 1 standard error of P-limited and P-replete *Tisochyrsis lutea* cultures fed to a) nauplii and b) copepodites during each experiment (25, 28, 32°C). Stoichiometry of *T. lutea* cultures fed to nauplii varied with treatment (P-limited or P-replete), but not between experiments ($F_{1,12}$ = 20.82, p < 0.001). Stoichiometry of *T. lutea* cultures fed to copepodites varied with treatment ($F_{1,12}$ = 53.35, p < 0.001) and temperature ($F_{1,12}$ = 6.41, p = 0.008). This was due to P-limited cultures having lower C:P ratios than expected in the 28°C experiment on days 4 and 5 ($F_{1,12}$ = 6.32, p = 0.008).

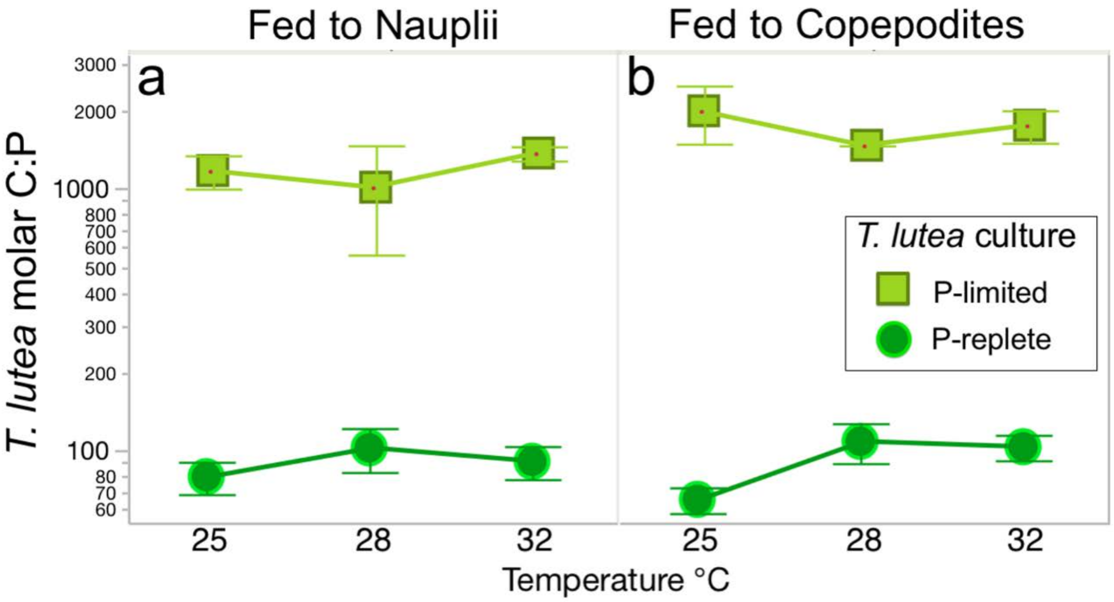

Fig. S3. The relationship between nauplii and copepodite growth and *T. lutea* C:P stoichiometry at 25°C, 28°C and 32°C. Nauplii specific growth rates (d^{-1}) and individual dry weight were negatively related to increasing C:P stoichiometry (Growth: $r^2 = 0.253$, $p = 0.019$; Dry weight: $r^2 = 0.195$, $p = 0.038$). However copepod specific growth rates (d^{-1}) and body mass were unrelated to the C:P stoichiometry of their food.

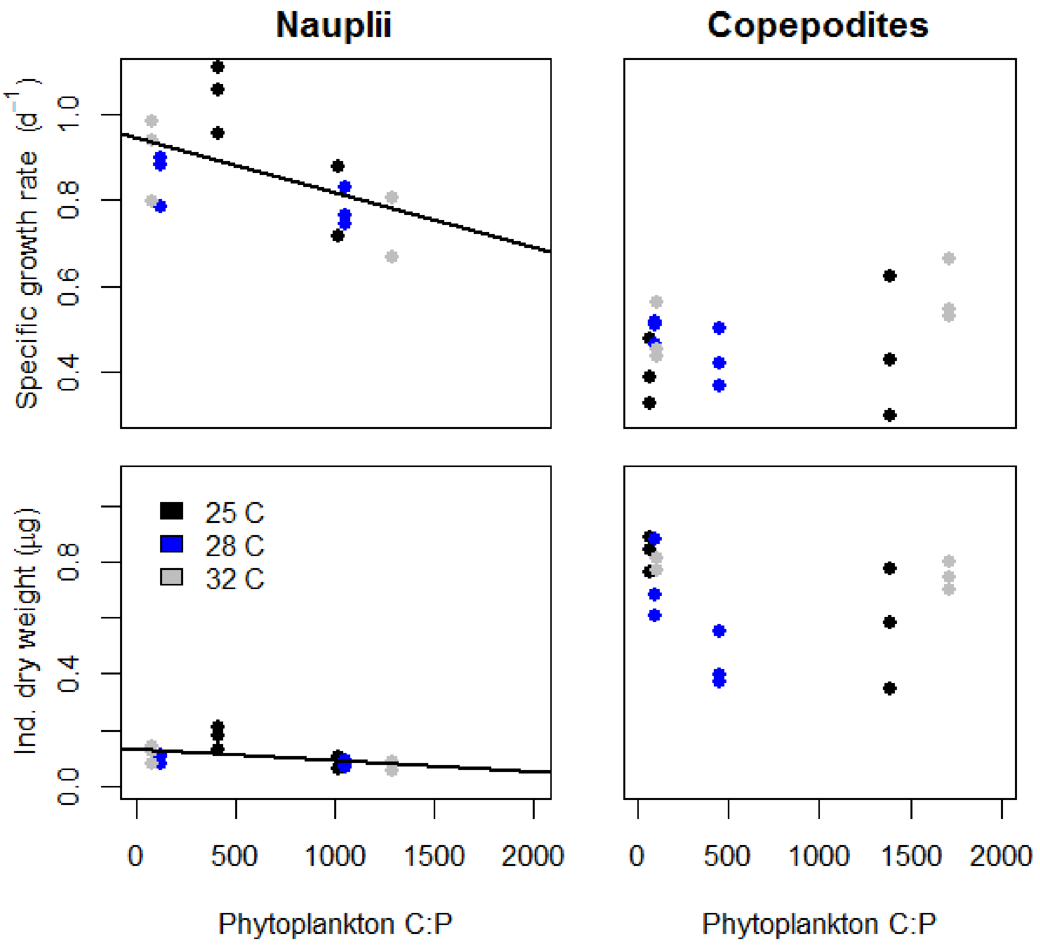

Supplement: Supplementary file 1 — Supplementary material 1 (PDF 182 kb) [file 442_2018_4183_MOESM1_ESM.pdf]
